# ApoCIII enrichment in HDL impairs HDL-mediated cholesterol efflux capacity

**DOI:** 10.1038/s41598-017-02601-7

**Published:** 2017-05-24

**Authors:** Mengdie Luo, Aiying Liu, Shuai Wang, Tianle Wang, Die Hu, Sha Wu, Daoquan Peng

**Affiliations:** Department of Cardiovascular Medicine, The Second Xiangya Hospital, Central South University, Changsha, Hunan China

## Abstract

Apolipoprotein CIII (apoCIII) has been reported to be tightly associated with triglyceride metabolism and the susceptibility to coronary artery disease (CAD). Besides, apoCIII has also been found to affect the anti-apoptotic effects of HDL. However, the effect of apoCIII on HDL-mediated cholesterol efflux, the crucial function of HDL, has not been reported. A hospital-based case-control study was conducted to compare the apoCIII distribution in lipoproteins between CAD patients and nonCAD controls and to explore the relationship between HDL-associated apoCIII (apoCIII_HDL_) and HDL-mediated cholesterol efflux. One hundred forty CAD patients and nighty nine nonCAD controls were included. Plasma apoCIII, apoCIII_HDL_ and cholesterol efflux capacity was measured. The apoCIII_HDL_ ratio (apoCIII_HDL_ over plasma apoCIII) was significantly higher in CAD patients than that in control group (0.52 ± 0.24 vs. 0.43 ± 0.22, P = 0.004). Both apoCIII_HDL_ and apoCIII_HDL_ ratio were inversely correlated with cholesterol efflux capacity (r = −0.241, P = 0.0002; r = −0.318, P < 0.0001, respectively). Stepwise multiple regression analysis revealed that the apoCIII_HDL_ ratio was an independent contributor to HDL-mediated cholesterol efflux capacity (standardized β = −0.325, P < 0.001). This study indicates that the presence of apoCIII in HDL may affect HDL-mediated cholesterol efflux capacity, implying the alternative role of apoCIII in the atherogenesis.

## Introduction

Many epidemiological studies demonstrated that high density lipoprotein (HDL)-cholesterol (HDL-C) was reduced in patients with coronary artery disease (CAD) and that HDL-C level was inversely correlated with CAD incidence^1, 2^, which led to the development of raising HDL-C as a therapeutic approach to prevent CAD. However, results from randomized controlled trails^3, 4^ and Mendelian randomization studies^5^ failed to prove the protective effects of HDL-C. Scientific interest has been gradually shifted from raising HDL-C to improving HDL function. HDL protects against CAD via multiple mechanisms and the most important one is reverse cholesterol transport (RCT)^6^. Previous study showed that cholesterol efflux from macrophages, a crucial step of RCT, had a strong inverse association with carotid intima-media thickness and CAD likelihood independent of HDL-C level^7^. Our observation also confirmed the reverse correlation between cholesterol efflux and carotid intima-media thickness in patients with chronic kidney diseases^8^. Recent prospective cohort study revealed that HDL-mediated cholesterol efflux capacity, instead of HDL-C level, was an independent predictor of CAD incidence^9^. Unraveling the molecular determinants of HDL-mediated cholesterol efflux capacity may help the development of therapeutic interventions to improve HDL function^10^. Our previous work showed that tryptophan oxidation in apoAI was responsible for the myeloperoxidase-mediated loss of apoAI function and impairment of cholesterol efflux capacity^11^. Animal experiments also revealed that increased proinflammatory protein components in HDL, such as serum amyloid A (SAA), attenuated HDL cholesterol efflux capacity and that SAA genetic ablation restored HDL function^12, 13^.

Apolipoprotein CIII (apoCIII), a critical modulator of triglyceride metabolism, mainly presents on HDL particles ﻿and﻿ triglyceride-rich lipoproteins (TRLs) including chylomicron (CM) and very low-density lipoprotein (VLDL)^14^. Genetic studies revealed that loss-of-function (LOF) mutations in the apoCIII gene were associated with reduced triglyceride concentration and decreased CAD incidence^15, 16^. Another prospective cohort study showed that the percentage of apoCIII-containing HDL particle was correlated with CAD risk^17^. *In vitro* experiments suggested that the deterioration of the HDL anti-apoptotic effects in CAD patients was associated with HDL proteome remodeling including apoCIII increase in HDL particles^18^. However, the effect of apoCIII on HDL-mediated cholesterol efflux has not been reported. In this study, we found that apoCIII_HDL_ and the apoCIII_HDL_ ratio were increased in CAD patients compared to controls and that the apoCIII_HDL_ ratio was an independent contributor to HDL-mediated cholesterol efflux capacity. These findings indicate that apoCIII redistribution may result in HDL dysfunction and CAD progression.

## Results

### Characteristics of subjects

Demographic and biochemical characteristics of participants were shown in Table 1. FFA and hsCRP were significantly higher, while TC and HDL-C, LDL-C, apoAI were significantly lower in CAD patients compared to nonCAD controls. The percentage of diabetes, statin therapy and smoking was significantly higher in CAD group with comparison to the controls. Other parameters had no statistically significant differences between two groups.

**Table 1:** Baseline characteristics of all the subjects.

Variables	CAD (n = 140)	nonCAD (n = 99)	P
Male (%)	66.4	54.5	NS
Age (years)	63.10 ± 8.42	62.81 ± 8.01	NS
BMI (kg/m^2^)	24.43 ± 3.72	24.12 ± 3.02	NS
TG (mmol/L)	1.49 ± 0.87	1.47 ± 0.88	NS
TC (mmol/L)	3.75 ± 0.92	4.16 ± 1.02	0.002
HDL-C (mmol/L)	0.99 (0.85–1.19)	1.16 (0.91–1.33)	0.002
LDL-C (mmol/L)	2.27 ± 0.76	2.56 ± 0.81	0.006
apoAI (g/L)	1.08 ± 0.21	1.17 ± 0.24	0.002
apoB (g/L)	0.84 ± 0.25	0.89 ± 0.26	NS
hsCRP (mg/L)	4.44 (1.21–12.33)	2.27 (0.96–6.62)	0.032
FFA (mmol/L)	0.46 (0.31–0.69)	0.40 (0.25–0.56)	0.014
Diabetes (%)	24.29	7.07	0.0004
Statin (%)	59.29	11.11	<0.0001
Smoking (%)	47.86	28.28	0.003
Drinking (%)	25.71	15.15	NS

Values are expressed as mean ± SD or median (interquartile range). CAD indicates coronary artery disease; BMI, body mass index; TG, triglyceride; TC, total cholesterol; HDL-C, high density lipoprotein-cholesterol; LDL-C, low density lipoprotein-cholesterol; apoAI, apolipoprotein AI; apoB, apolipoprotein B; hsCRP, high sensitivity C reactive protein; FFA, free fatty acid.

### ApoCIII levels in CAD group

**Figure 1:**
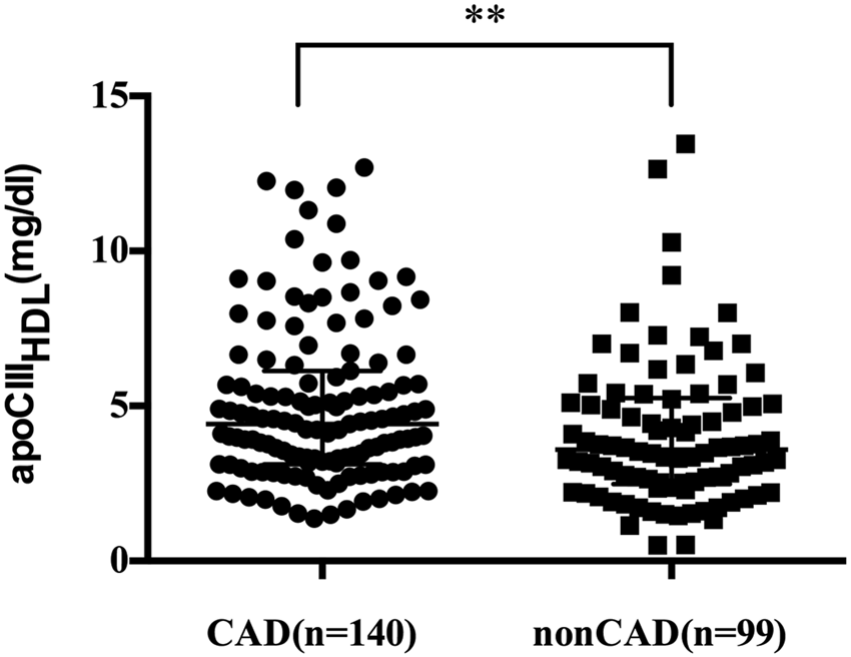
ApoCIII_HDL_ from CAD (n = 140) and nonCAD (n = 99) patients. Data are expressed as median ± interquartile range. ApoCIII_HDL_ indicates apolipoprotein CIII in apoB-depleted plasma; apoB, apolipoprotein B; CAD, coronary artery disease. **P < 0.01.

**Figure 2:**
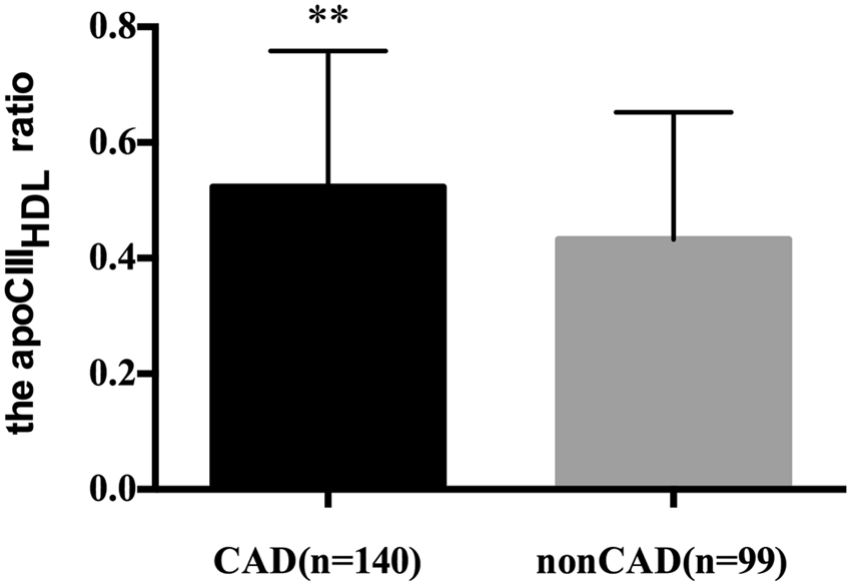
The apoCIII_HDL_ ratio (apoCIII_HDL_ over plasma apoCIII) between CAD (n = 140) and nonCAD (n = 99) patients. Data are expressed as mean ± SD. ApoCIII_HDL_ indicates apolipoprotein CIII in apoB-depleted plasma; apoB, apolipoprotein B; CAD, coronary artery disease. **P < 0.01.

Plasma apoCIII and apoCIII_HDL_ were measured via ELISA method. No significant difference was seen in plasma apoCIII between CAD group [9.40 (6.56, 12.28) mg/dl] and non-CAD group [9.42 (6.80, 13.86) mg/dl, P = 0.575]. Both apoCIII_HDL_ and the apoCIII_HDL_ ratio, the quotient of apoCIII_HDL_ over plasma apoCIII, were significantly higher in CAD group than that in nonCAD group [apoCIII_HDL_: 4.42 (3.12, 6.13) mg/dl vs. 3.59 (2.48, 5.26) mg/dl, P = 0.007, Fig. 1; the apoCIII_HDL_ ratio: 0.52 ± 0.24 vs. 0.43 ± 0.22, P = 0.004, Fig. 2]. No significant differences in plasma apoCIII, apoCIII_HDL_ and apoCIII_HDL_ ratio were observed between statin users and non-statin users, diabetes patients and non-diabetes patients, smokers and non-smokers (Table 2).

**Table 2:** Comparison of apoCIII concentration and HDL-mediated cholesterol efflux capacity between different groups.

	DM			Statin			Smoking		
	Yes	No	P	Yes	No	P	Yes	No	P
Plasma apoCIII (mg/dl)	10.61 (7.73–12.95)	9.20 (6.34–13.08)	0.21	9.60 (6.77–12.93)	9.04 (6.53–13.50)	0.44	9.20 (6.62–14.59)	9.46 (6.56–12.86)	0.99
apoCIII_HDL_ (mg/dl)	4.87 (3.07–5.92)	3.92 (2.76–5.69)	0.21	4.56 (3.33–6.61)	3.65 (2.60–5.35)	0.08	3.79 (2.74–5.69)	4.15 (3.04–5.69)	0.32
apoCIII_HDL_ ratio	0.474 ± 0.189	0.488 ± 0.241	0.98	0.523 ± 0.256	0.461 ± 0.213	0.15	0.462 ± 0.226	0.501 ± 0.237	0.12
CEC (%)	12.2 ± 3.2	13.6 ± 2.6	0.02	12.8 ± 2.9	13.8 ± 2.6	0.03	13.3 ± 2.9	13.5 ± 2.6	0.60

DM indicates diabetes mellitus; apoCIII, apolipoprotein C-III; apoCIII_HDL_, apolipoprotein CIII in apoB-depleted plasma; CEC, cholesterol efflux capacity.

### Correlation analysis of apoCIII and triglyceride

**Figure 3:**
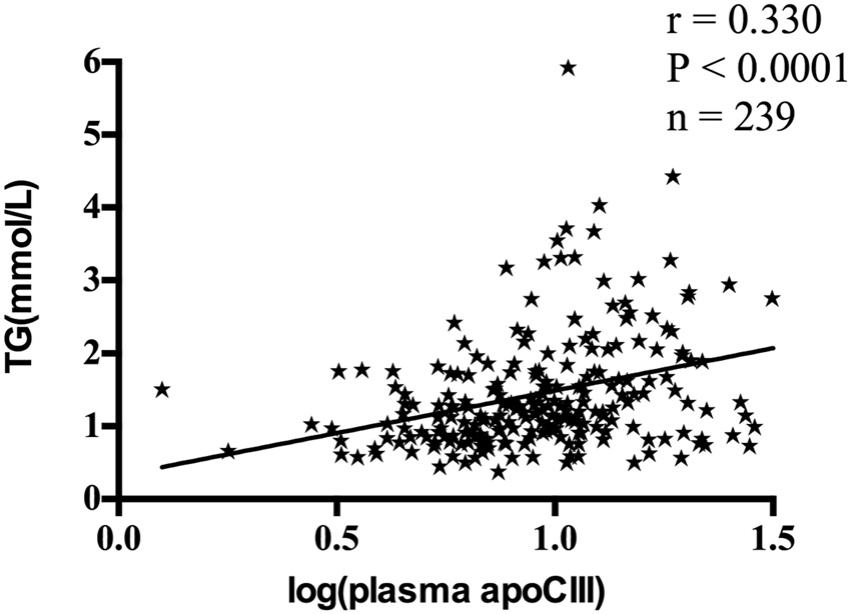
The correlation between plasma apoCIII (log transformed) and triglyceride in all the subjects (n = 239). ApoCIII indicates apolipoprotein CIII; TG, triglyceride.

To examine the relationship between apoCIII and triglyceride, correlation analyses were performed. As previously mentioned, plasma apoCIII and apoCIII_HDL_ were log-transformed to be normally distributed. The results showed that plasma apoCIII concentration was positively associated with plasma triglyceride levels (r = 0.330, P < 0.0001, Fig. 3). Within-group analysis showed that the correlation between plasma apoCIII and triglyceride remained significant in CAD and nonCAD groups (Table 3). However, no significant relationship was found between apoCIII_HDL_ (log transformed) and plasma triglyceride in within-group analysis or combined analysis (Table 4).

**Table 3:** Pearson’s correlation analyses between log-transformed plasma apoCIII and clinical and biochemical parameters.

	All subjects (n = 239)		CAD (n = 140)		nonCAD (n = 99)	
	r	P	r	P	r	P
TG	0.330	<0.0001	0.371	<0.0001	0.276	0.008
TC	0.208	0.002	0.261	0.002	0.136	0.193
LDL-C	0.207	0.002	0.264	0.002	0.127	0.224
apoB	0.249	0.0002	0.282	0.001	0.195	0.065
hsCRP (log-transformed)	0.059	0.384	0.016	0.853	0.136	0.198

CAD indicates coronary artery disease; TG, triglyceride; TC, total cholesterol; LDL-C, low-density lipoprotein-cholesterol; apoB, apolipoprotein B; hsCRP, high-sensitivity C reactive protein.

**Table 4:** Pearson’s correlation analyses between log-transformed apoCIII_HDL_ and clinical and biochemical parameters.

	All subjects (n = 239)		CAD (n = 140)		nonCAD (n = 99)	
	r	P	r	P	r	P
plasma apoCIII (log-transformed)	0.607	<0.0001	0.688	<0.0001	0.555	<0.0001
TG	−0.025	0.705	0.103	0.234	−0.064	0.542
CEC	−0.241	0.0002	−0.158	0.068	−0.247	0.018
CK	0.163	0.022	0.214	0.021	−0.077	0.495
UA	0.186	0.005	0.224	0.01	0.202	0.051
HDL-C (log-transformed)	0.083	0.214	0.042	0.627	0.199	0.055
hsCRP (log-transformed)	0.041	0.547	−0.097	0.273	0.158	0.135

CAD indicates coronary artery disease; apoCIII, apolipoprotein CIII; TG, triglyceride; CEC, cholesterol efflux capacity; CK, Creatine phosphokinase; UA, uric acid; HDL-C, high density lipoprotein-cholesterol; hsCRP, high sensitivity C reactive protein.

### Correlation analysis of apoCIII and other parameters associated with CAD

Plasma apoCIII (log transformed) was positively correlated with TC (r = 0.208, P = 0.002) and LDL-C (r = 0.207, P = 0.002) and apoB (r = 0.249, P = 0.0002) in all the subjects (Table 3). The correlation remained significant in patients with CAD but not in nonCAD subjects (Table 3). ApoCIII_HDL_ (log transformed) was positively correlated with plasma apoCIII (r = 0.607, P < 0.0001), but negatively correlated with cholesterol efflux capacity (r = −0.241, P = 0.0002) in all the subjects (Table 4). In order to investigate the relationship between apoCIII and inflammation, correlation analysis of apoCIII to hsCRP, a typical inflammation marker, was performed. The results showed that plasma apoCIII, apoCIII_HDL_ and the apoCIII_HDL_ ratio did not have significant relationship with hsCRP (Tables 3 and 4).

### HDL-mediated Cholesterol efflux capacity

**Figure 4:**
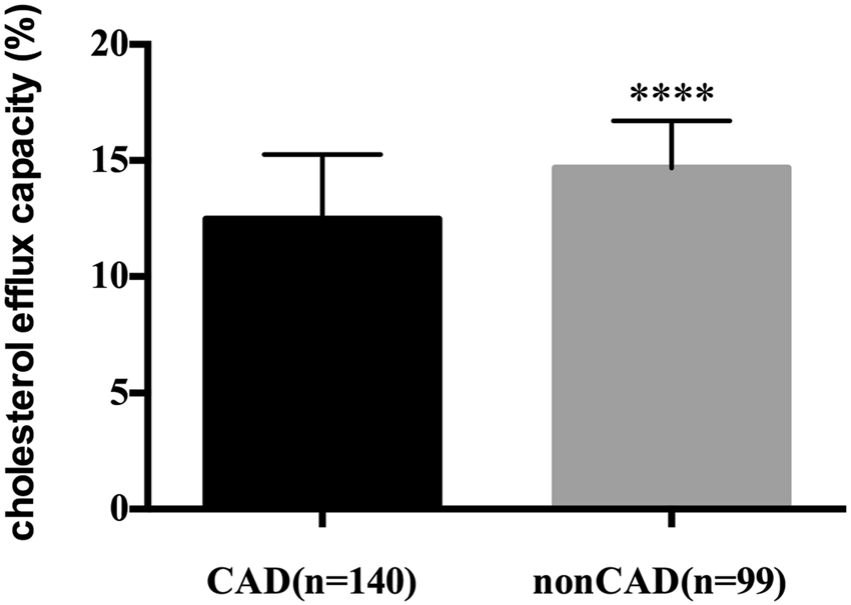
Cholesterol efflux capacity in CAD (n = 140) and nonCAD (n = 99) patients. Data are expressed as mean ± SD. CAD indicates coronary artery disease. ****P < 0.0001.

In order to measure HDL-mediated cholesterol efflux capacity, apoB-depleted plasma obtained by heparin-manganese method was adopted. Cholesterol efflux capacity was significantly lower in CAD patients than that in nonCAD patients (12.5 ± 2.8% vs. 14.7 ± 2.0%, P < 0.0001, Fig. 4). To investigate the impacts of statin use, smoking and diabetes status on the cholesterol efflux capacity, subjects were divided into groups accordingly. We found that cholesterol efflux capacity in subjects without diabetes was significantly higher than that in subjects with diabetes (13.6 ± 2.6% vs. 12.2 ± 3.2%, P = 0.021, Table 2). Besides, non-statin users also present significantly higher cholesterol efflux capacity than statin users (13.8 ± 2.6% vs. 12.8 ± 2.9%, P = 0.026, Table 2). However, smoking had no effects on cholesterol efflux capacity (smokers vs. non-smokers 13.3 ± 2.9% vs. 13.5 ± 2.6%, P = 0.597, Table 2).

### Relationship between apoCIII and cholesterol efflux capacity

**Figure 5:**
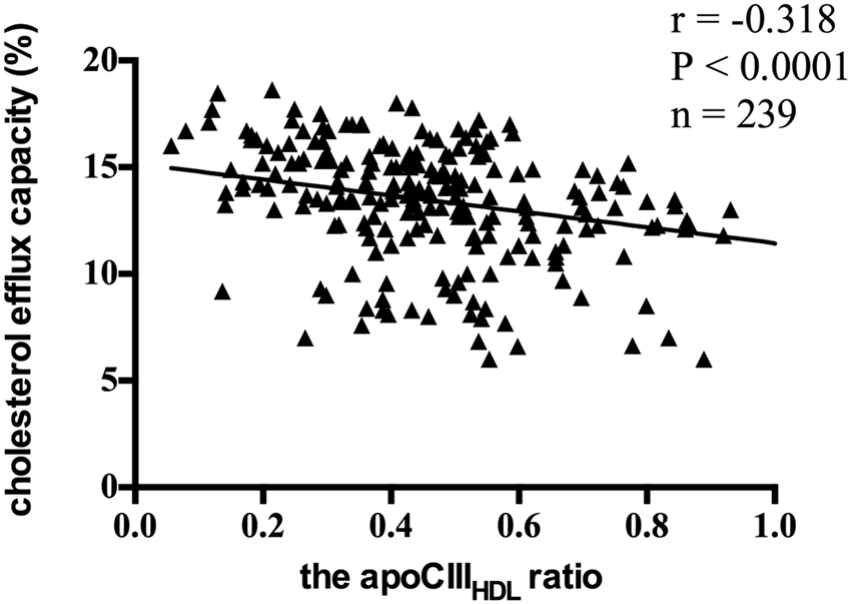
The correlation of the apoCIII_HDL_ ratio and cholesterol efflux capacity in all the subjects (n = 239). ApoCIII_HDL_ indicates apolipoprotein CIII in apoB-depleted plasma.

No research has been reported to investigate the relationship between HDL-associated apoCIII and HDL-mediated cholesterol efflux capacity. In our research, cholesterol efflux capacity was inversely correlated with BMI (r = −0.147, P = 0.028), log-transformed apoCIII_HDL_ (r = −0.241, P = 0.0002), the apoCIII_HDL_ ratio (r = −0.318, P < 0.0001, Fig. 5) and log-transformed hsCRP (r = −0.248, P = 0.0002). The positive correlation with HDL-C (r = 0.443, P < 0.0001) and apoAI (r = 0.541, P < 0.0001) has been validated (Table 5). In order to exclude confounding effects of HDL-C on the correlation of HDL-mediated cholesterol efflux capacity to apoCIII_HDL_ and the apoCIII_HDL_ ratio, partial correlation analysis was performed after controlling HDL-C level. The inverse correlation remained significant for apoCIII_HDL_ (r = −0.314, P < 0.001) and for apoCIII_HDL_ ratio (r = −0.423, P < 0.001). Besides, in order to check for the effects of inflammation, partial correlation analysis was conducted after controlling hsCRP. HDL-mediated cholesterol efflux capacity was still inversely correlated with apoCIII_HDL_ (r = −0.230, P = 0.001) and for apoCIII_HDL_ ratio (r = −0.348, P < 0.001) after controlling hsCRP.

**Table 5:** Pearson’s correlation analyses between HDL-mediated cholesterol efflux capacity and clinical and biochemical parameters.

	All subjects (n = 239)		CAD (n = 140)		nonCAD (n = 99)	
	r	P	r	P	r	P
BMI	−0.147	0.028	−0.160	0.064	−0.103	0.328
plasma apoCIII (log-transformed)	0.069	0.302	0.023	0.791	0.134	0.204
apoCIII_HDL_ (log-transformed)	−0.241	0.0002	−0.158	0.068	−0.247	0.018
apoCIII_HDL_ ratio	−0.318	<0.0001	−0.199	0.021	−0.409	<0.0001
hsCRP (log-transformed)	−0.248	0.0002	−0.205	0.019	−0.237	0.025
apoAI	0.541	<0.0001	0.585	<0.0001	0.442	<0.0001
HDL-C (log-transformed)	0.443	<0.0001	0.448	<0.0001	0.373	0.0003

CAD indicates coronary artery disease; BMI, body mass index; apoCIII, apolipoprotein CIII; hsCRP, high sensitivity C reactive protein; apoAI, apolipoprotein AI; HDL-C, high-density lipoprotein-cholesterol.

### Multiple regression analysis

In addition to HDL-C and hsCRP, other factors may also confound the relationship between apoCIII distribution in HDL and HDL-mediated cholesterol efflux. A stepwise multiple regression model was fitted after adjustment for diabetes status, statin use and the variables that were significantly correlated with HDL-mediated cholesterol efflux capacity in univariate analysis, including BMI, apoCIII_HDL_ ratio, hsCRP, apoAI and HDL-C. Log-transformed values were used for the variables skewed distributed, including hsCRP and HDL-C. The regression analysis revealed that the apoCIII_HDL_ ratio, apoAI and diabetes status were independent risk factors for HDL-mediated cholesterol efflux capacity (Table 6).

**Table 6:** Stepwise multiple regression analysis detecting independent contributors to HDL-mediated cholesterol efflux capacity in all the subjects.

Factors	β	Standardized β	P
BMI	<0.001	−0.058	0.274
apoCIII_HDL_ ratio	−0.038	−0.325	<0.001
apoAI	0.067	0.558	<0.001
hsCRP (log-transformed)	−0.002	−0.053	0.348
HDL-C (log-transformed)	−0.016	−0.065	0.541
Diabetes	−0.012	−0.159	0.003
Statin	−0.005	−0.097	0.065

BMI indicates body mass index; apoCIII_HDL_ indicates apolipoprotein CIII in apoB-depleted plasma; apoAI, apolipoprotein AI; hsCRP, high sensitivity C reactive protein; HDL-C, high density lipoprotein-cholesterol.

R square: 0.452, P < 0.001.

## Discussion

In this study, we found that apoCIII_HDL_ and the apoCIII_HDL_ ratio were significantly higher in CAD patients compared to nonCAD controls. Besides, they were inversely correlated with HDL-mediated cholesterol efflux capacity. Moreover, increased apoCIII_HDL_ ratio was an independent contributor to impaired HDL-mediated cholesterol efflux capacity.

Our experiments showed that plasma apoCIII was significantly correlated with triglyceride, which was consistent with the established role of apoCIII in triglyceride metabolism^14^. Recent genetic epidemiological studies found that LOF mutation in apoCIII gene was associated with decreased triglyceride level and reduced CAD risk^15, 16^. On the other hand, gain of function mutant of apoCIII gene, T-455C variant, was found to be associated with elevated apoCIII and triglyceride levels and increased CAD probability in metabolic syndrome (MS) patients^19^. However, disappointing results from randomized controlled trials of fenofibrate and omega-3 fatty acids challenged the role of triglyceride in CAD risk^20, 21^. These controversial findings gave rise to the hypothesis that triglyceride-related effects on CAD incidence might be mediated by the direct atherogenic effects of apoCIII^22^. However, our results did not show significant difference in plasma apoCIII between CAD and nonCAD group, but apoCIII in HDL particles was significantly increased in CAD patients compared to controls, which was in accordance with previous studies^23, 24^. These findings indicated that apoCIII distribution in lipoproteins, instead of total apoCIII concentration, might paly an important role in CAD propensity. In circulation, apoCIII mainly resides on the surface of lipoproteins and apoCIII is transferable between TRLs and HDL^14, 23^. In our study, the apoCIII_HDL_ ratio was significantly higher in CAD group than that in nonCAD group, which indicated that more apoCIII was transferred from apoB-containing lipoproteins to HDL in CAD patients. Previous study revealed that two HDL subtypes, HDL with apoCIII and HDL without apoCIII, were oppositely associated with CAD risk^17^. Besides, per standard deviation increase of apoCIII-containing HDL particle was associated with an 18% higher risk for CAD^17^. But the mechanism by which apoCIII damaged the protective effects of HDL on CAD risk was still not clearly defined. Current study suggested that accumulation of apoCIII in HDL particles might impair HDL-mediated cholesterol efflux capacity, the crucial athero-protective effect of HDL. HDL anti-apoptotic property was also attenuated by the presence of apoCIII in HDL as demonstrated by Riwanto *et al*.^18^. Being a reasonable index of relative enrichment of apoCIII in HDL, the apoCIII_HDL_ ratio might be regarded as a significant biomarker for HDL dysfunction and CAD susceptibility. Results from Chin-Shan Community Cardiovascular Cohort Study suggested that HDL-apoCIII to VLDL-apoCIII ratio was a reliable marker to predict CAD^23^, which was in agreement with our findings. But the measurement of HDL-apoCIII to VLDL-apoCIII ratio involved sophisticated lipoprotein isolation and therefore it was less clinically applicable.

Inflammation was related to the alteration of HDL function. Our early work showed that myeloperoxidase-mediated oxidation could modify tryptophan in apoAI and thus impair the cholesterol acceptor activity of apoAI^11^. In addition to the apoAI modification, HDL might be subjected to structure remodeling following inflammation. For example, increased SAA was found in HDL particle in response to the acute inflammation stress^12, 13^. Increased SAA in HDL could interact with proteoglycans on the cell surface and lead to HDL trapping in the extracellular matrix, thereby disrupting HDL-mediated cholesterol efflux^12, 13^. ApoCIII was reported to be involved in inflammation response. Increasing apoCIII content was accompanied by increasing apoCIII sialylation and apoCIII sialylation further promoted the proinflammatory properties of apoCIII^25^. ApoCIII could induce the adhesion of monocytes to endothelial cells and enhance the pro-inflammatory properties of VLDL and LDL particles^26, 27^. It was still unclear how the enrichment of apoCIII in HDL affected HDL-mediated cholesterol efflux. In this study, the correlation between apoCIII_HDL_ ratio and HDL-mediated cholesterol efflux remained significant after controlling for CRP, a marker of inflammation, suggesting that the effect of apoCIII on HDL-mediated cholesterol efflux might be independent of its pro-inflammatory effects.

Cholesterol efflux is a process in which apoAI or HDL accepts cholesterol from peripheral cells via several membrane transporters: ATP-binding cassette A1 (ABCA1), ATP-binding cassette G1 (ABCG1) and scavenger receptor family B type I (SR-BI)^28^. ApoAI accepts cellular cholesterol and phospholipids transported by ABCA1 to form nascent HDL particles^29^. HDL particles depend on ABCG1 and SR-BI to accept peripheral cellular cholesterol and mediate cholesterol efflux. SR-BI can mediate bi-directional cholesterol flux, including cholesterol efflux from peripheral cells to HDL and cholesterol uptake from HDL by hepatocytes^30^. ApoCIII can bind to hepatic SR-BI and inhibit hepatocytes to intake cholesterol from HDL and LDL^14, 31^. Based on these findings, we hypothesized that elevated apoCIII in HDL could competitively bind to SR-BI and interrupt the cholesterol unloading from HDL, thereby impairing HDL-mediated cholesterol efflux.

In the current study, patients with diabetes exhibited significantly lower cholesterol efflux capacity compared to non-diabetic subjects. The impairment of HDL-mediated cholesterol efflux capacity was potentially related to the increased HDL-apoCIII level in diabetic patients, although the increase of HDL-apoCIII did not reach statistical significance in the study. But more evidence was needed to elucidate the definite role of HDL-apoCIII in the impairment of cholesterol efflux capacity in diabetic subjects. Other factors have been reported to contribute to the reduced cholesterol efflux, including the increased apoAI nitrosylation^32^ and elevated SAA concentration^33^ in HDL particles from DM patients. Another interesting finding was that statin users had significantly lower cholesterol efflux capacity compared to the non-statin users. This phenomenon might be confounded by the higher CAD prevalence in statin users (88.3%) compared to that in non-statin users (39.3%) as CAD patients usually exhibited impaired cholesterol efflux capacity. In addition, previous study showed that HDL-apoCIII was elevated after statin treatment in CAD patients^24^ and this might help explain the reduced cholesterol efflux capacity in statin users. However, the effects of statin therapy on HDL-mediated cholesterol efflux were inconsistent in different researches^28^. Concerning the positive correlation of HDL-apoCIII and uric acid, no clear evidence has been found. The detailed effects of uric acid on apoCIII distribution and CAD progression still need more investigation.

To the best of our knowledge, no experiments have been conducted to observe the relationship between HDL-associated apoCIII and HDL-mediated cholesterol efflux capacity in the CAD progression. In the current study, we showed that the apoCIII_HDL_ ratio was an independent contributor to HDL-mediated cholesterol efflux capacity, which provided evidence for the role of apoCIII in HDL dysfunction and a possible explanation for the direct atherogenic effects of apoCIII. However, apoCIII may be just a bystander of other constituents remodeling of HDL in CAD progression. Whether the correlation of HDL-associated apoCIII and HDL dysfunction is causative is still not clear. Further *in vitro* and animal studies are warranted to investigate the causal relationship between apoCIII and HDL-mediated cholesterol efflux.

## Methods

### Subjects

We recruited 239 subjects from the Department of Cardiovascular Medicine of Second Xiangya Hospital, Central South University. To evaluate the relationship of HDL-apoCIII with cholesterol efflux capacity, we studied 2 groups: CAD group (n = 140) and nonCAD group (n = 99). In this study, CAD meant acute coronary syndrome (ACS). ACS included ST-segment elevated myocardial infarction (STEMI), non ST-segment elevated myocardial infarction (NSTEMI) and unstable angina. ACS diagnosis was based on the clinical symptoms and signs, ischemic electrocardiographic abnormalities, and coronary angiography showing ≥ 50% stenosis in at least one main coronary artery. The exclusion criteria included: a history of renal failure, chronic hepatic diseases, high fever, or bacterial/viral infection, autoimmune disease, arthritis, malignancies, severe diabetes and hypertension, and other severe medical illnesses. All the subjects provided written informed consent. The study was approved by the Medical Ethics Committee of the Xiangya Second Hospital of Central South University and was conducted in accordance with approved guidelines and regulations.

### Clinical and biochemical measurements

Patient information, including age, gender, smoking and drinking history, and statin therapy history, was recorded. The details of anthropometric measurements (weight, height, body mass index) were assessed after overnight fasting for at least 10 hours. Peripheral blood samples were obtained from patients’ brachial veins. Subjects fasted for at least 10 hours before blood collection and then blood routine, urine routine, concentrations of lipid parameters, including total cholesterol (TC), triglyceride (TG), low-density lipoprotein cholesterol (LDL-C), HDL-C, apoAI, apoB, free fatty acid (FFA), were evaluated via standard laboratory procedures. Concentrations of high-sensitivity C-reactive protein (hsCRP) were measured with a latex particle, enhanced immunoturbidimetric assay. For the subsequent experiments, fresh plasma was obtained by centrifugation at 3000 r/min at 4 °C for 10 minutes. The plasma was aliquoted and stored at −80 °C freezer until analysis.

### ApoB-depleted plasma preparation

According to previous experiments^34^, 540 ul heparin sodium solution (280 mg/ml, Aladdin, H104201) and 10 ml manganese chloride solution (1.06 mol/L, Aladdin, M112542) was mixed. 1 ml plasma was incubated for 30 minutes at 4 °C with 100 ul mixed solution, and then centrifuged at 1500 g for 30 minutes. Supernatant was collected. If supernatant was still turbid (especially samples from patients with hypertriglyceridemia), plasma was centrifuged at 12000 g for 10 minutes again. Previous study revealed that heparin sodium/manganese chloride precipitation had no effects on HDL size as well as cholesterol efflux measurement^35^, and therefore this method was chosen to prepare apoB-depleted plasma in the study.

### Measurement of plasma apoCIII and apoCIII_HDL_

Plasma apoCIII and apoCIII_HDL_ was determined by a sandwich enzyme-linked immunosorbent assay (ELISA). ApoCIII_HDL_ was referred to as apoCIII in apoB-depleted plasma according to previous literature^19, 36^. Commercial ApoCIII ELISA kits (Abcam, ab154131) were used to quantify the concentrations. All the measurement of plasma apoCIII and apoCIII_HDL_ were performed in duplicate for each sample. The coefficient of variation for intra- and inter-assay variation was 2.9% and 3.4%, respectively.

### Cholesterol efflux capacity measurement

Cholesterol efflux experiments were performed according to established procedures^11, 37^. THP-1 human monocytes (ATCC) were grown in RPMI1640 medium (Gibco, 22400089), supplemented with 10% heat-inactivated FBS, 1% penicillin/streptomycin until differentiation into macrophages by the addition of phorbol myristate acetate (100 ng/ml, Sigma, P1585). Subsequently, differentiated THP-1 macrophages were loaded with 50 ug/mL acetylated LDL (Peking Union-Biology Co.Ltd) and 1 uCi/mL [3 H] cholesterol for 24 hours. Macrophages were then washed twice with PBS (Gibco, 10010023) and equilibrated for 24 hours in RPMI1640 medium containing 2% bovine serum albumin. Cells were then washed with PBS again and apoB-depleted plasma from individual patients was diluted in medium (2.5%, vol/vol). After 16 hours, the supernatant was collected and centrifuged to remove cellular debris. The cells were washed twice with PBS, and then incubated for at least 30 min at room temperature with 0.1 mol/L NaOH solution. The radioactivity within the supernatant and cells was determined by liquid scintillation counting. Wells incubated with RPMI1640 but without added apoB-depleted plasma were used as blanks, and these values were subtracted from the respective experimental values. Efflux is given as the percentage of counts recovered from the medium in relation to the total counts present on the plate (sum of medium and cells). All efflux experiments were performed in duplicate for each sample with intra- and inter-assay coefficients of variation of 4.1% and 7.9%, respectively.

### Statistical analysis

Statistical analysis was performed with Statistical Package for Social Sciences version 22.0 and plots were made with GraphPad Prism V.6.0 (GraphPad Software, Inc, La Jolla, California, USA). Clinical data are expressed as mean ± standard deviation (normally distributed continuous data) or median with interquartile range (skewed distributed continuous data). Comparisons between categorical data were performed with Chi Squared tests, while continuous variables were assessed by unpaired t test (for normal distribution) or nonparametric test (for skewed distribution). To evaluate the associations between variables, Pearson correlation analysis was used. Stepwise multiple linear regression analysis was performed to determine the variables with independent significant association with cholesterol efflux capacity. These variables included all potential ones that might have significant relationship with cholesterol efflux capacity in univariate analyses. In the correlation and regression analysis, logarithmatic-transformed values were used for the variables skewed distributed. A two tailed P value < 0.05 was considered statistically significant.
